# Repeatability of a Commercially Available ELISA Test for Determining the Herd-Level *Salmonella enterica* subsp. *enterica* Serovar Dublin Status in Dairy Herds Using Bulk Milk

**DOI:** 10.3389/fvets.2020.00401

**Published:** 2020-07-10

**Authors:** Maryse Michèle Um, Marie-Hélène Castonguay, Khalie Mahamad Amine, Julie Giguère, Isabelle Morin, Simon Dufour

**Affiliations:** ^1^Department of Pathology and Microbiology, Faculty of Veterinary Medicine, University of Montreal, Saint-Hyacinthe, QC, Canada; ^2^Op+Lait, Faculty of Veterinary Medicine, University of Montreal, Saint-Hyacinthe, QC, Canada; ^3^Research Group in Epidemiology of Zoonoses and Public Health, Faculty of Veterinary Medicine, University of Montreal, Saint-Hyacinthe, QC, Canada; ^4^Lactanet, Sainte-Anne-de-Bellevue, QC, Canada

**Keywords:** *Salmonella* Dublin, ELISA, bulk milk, repeatability, freezing

## Abstract

An Enzyme-Linked Immunosorbent Assay (ELISA) is currently available for detection of antibodies against *Salmonella* Dublin in bovine milk. However, when used in a surveillance program, samples may undergo various storage conditions. The objective of this study was to estimate the repeatability of an ELISA test when used on fresh and frozen samples. Each of 845 bulk milk collected samples was subdivided into 3 aliquots and analyzed using PrioCHECK™ *Salmonella* Ab Bovine Dublin. ELISA percent positivity results (PP%) were compared between aliquots submitted to the initial analysis and a second analysis conducted 24 h later. The third aliquots were either preserved for 13–14 days (*n* = 413) or 25–28 days (*n* = 432) at −20°C prior to analysis and results were compared to the initial analysis. There was excellent concordance between the two initial values and with values obtained after 13–14 and 25–28 days-freezing. The corresponding concordance correlation coefficients were 0.96, 0.97, and 0.94, respectively. Bland-Altman plots showed differences of PP% of 0.1 percentage points on average between the initial and second fresh samples. Freezing for 13–14 and 25–28 days led to overestimation of the initial values by 0.1, and 0.4 percentage points, respectively. Regarding the classification of samples, greater disagreement was observed between 25 and 28 days-frozen and initial samples when using the cut-off 15% (kappa = 0.76) compared to 35% (kappa = 0.90). Our study showed that PrioCHECK™ has good repeatability and that frozen bulk milk samples could generate reliable results. However, the larger variability at lower PP% should be considered when setting up a threshold.

## Introduction

*Salmonella enterica* subsp. *enterica* serovar Dublin (*S*. Dublin) is an emerging zoonotic pathogen in dairy cattle and humans in Québec since 2011 (1). In infected herds, it leads to concerning levels of production losses but also causes enteric disease, pneumonia, septicemia especially among calves, and abortion in adult cattle (2, 3). It is an important infection in the dairy industry because it is adapted to cattle (4). Systemic invasion in carrier cows leads to production of specific antibodies which can be detected in milk samples (5, 6). Testing milk for antibodies to the *S*. Dublin O-antigen factors, notably by Enzyme-Linked Immunosorbent Assay (ELISA), therefore facilitates the identification of *S*. Dublin-infected herds (7).

The PrioCHECK™ *Salmonella* Ab Bovine Dublin, an indirect ELISA commercialized by Thermo Fischer Scientific, originates from the Danish Veterinary Laboratory (7). It is based on the detection of antibodies in cattle directed against *Salmonella* Dublin LPS O-antigens 1, 9, and 12. It reports the corrected optical density value of each milk sample as percent positivity (PP%). This test was designed for screening of individual cow milk. For a large-scale screening, however, bulk milk samples are simpler to collect and, thus, possibly more convenient samples than individual cow samples. For instance, the bulk milk samples that are already set aside on each milk collect, either for milk composition analyses for payment or for milk quality analyses, could possibly be used to determine the herd status for *S*. Dublin. However, in a *S*. Dublin surveillance program based on mandatory regular bulk milk ELISA analyses, samples may undergo various storage conditions, including freezing for many days. Currently, no information is available regarding the variability of that assay when performed on fresh or frozen samples.

The PrioCHECK™ and Danish-in-house milk based-ELISA have been evaluated in Northern European countries for their performance in diagnosing the *S*. Dublin herd-level status using bulk milk samples and seemed to have good performance when using the manufacturer's recommended cut-off PP% ≥ 35% (8). In the province of Québec (Canada), PrioCHECK™ is currently used in bulk milk for diagnosis of *S*. Dublin and results ≥ 15% are interpreted as positive by local authorities. The rationale for this lower threshold is that, when used on bulk milk samples in a population where prevalence of *S*. Dublin positive cows is expected to be low, the milk of a few *S*. Dublin positive cows may be diluted by those of numerous negative cows. Thus, lowering the threshold used for interpretation may help increasing the diagnostic sensitivity of the test, although the specificity would be decreased. So far, the repeatability of the test for determining if a sample is positive or negative using our provincial authorities' cut-off or manufacturer's suggested cut-off has not yet been published.

Consequently, the aim of this study was to assess the repeatability of the commercially available milk ELISA test in a context of a surveillance program by assessing the assay variability on fresh and frozen samples and the repeatability of the test for classifying a sample as positive or negative.

## Materials and Methods

### Study Design

This study was an experimental study conducted using bulk milk samples collected on 293 dairy farms participating in a larger observational cohort study aiming at validating a diagnostic strategy for detecting *S*. Dublin infected dairy herds. This research was approved by the Université de Montréal's Research Ethic Committee (18-Rech-1943).

#### Herds

The study was performed with samples from 47 dairy herds that have already tested positive to *S*. Dublin and 246 randomly selected dairy herds. The positive status of infected herds was confirmed with clinical signs, bacterial isolation and/or blood or milk serology. They were recruited by the Québec Ministry of Agriculture, Fisheries and Food (Ministère de l'agriculture, des pêcheries et de l'alimentation du Québec; MAPAQ) from a list of infected herds since 2014. The other herds were randomly recruited by the research team from a list of 954 dairy herds from three regions of the Québec Province (Montérégie, Centre-du-Québec, and Estrie) and provided by the provincial dairy producer association.

#### Data Collection

A total of 845 monthly bulk milk samples were collected from April to September 2019 within the mandatory provincial milk quality analysis scheme. Briefly, certified milk transporter aseptically sampled 50 to 60 ml from bulk tank milk of each dairy herd. Samples were kept refrigerated at 4°C during transportation and were transferred to a commercial laboratory (Lactanet) 24 to 48 h after the sampling. Each bulk milk sample was subdivided into 3 aliquots as presented in Figure S1. The first aliquots, preserved at 4°C, were submitted to the initial analysis in general within 7 days after arrival to the laboratory. The second aliquots kept at 4°C were analyzed 24 h following the initial analysis. The third aliquots were either preserved for 13 to 14 days (*n* = 413) or 25 to 28 days (*n* = 432) at −20°C prior to analysis.

### ELISA Assay

The samples were tested at the laboratory using the PrioCHECK™ *Salmonella* Ab Bovine Dublin, Ref. 7610640 (Thermo Fischer Scientific, USA). Antibodies against *S*. Dublin LPS O-antigens 1, 9, and 12 were detected from plates coated with the purified LPS isolated from *S*. Dublin. The analyses were carried out following the instructions of the manufacturer. The optical density of each well was measured at 450 nm. The ELISA results were interpreted using corrected OD_450_ values (sample OD minus negative control OD) and expressed as percent positivity (PP %), which is the ratio (%) of the sample corrected OD_450_ to the positive control corrected OD_450_ minus 10 according to Equation 1. The samples were initially classified as positive when PP% ≥ 15%, which is the Provincial authorities' used cut-off. Alternatively, a second classification using the PP% ≥ 35% manufacturer's cut-off was used for comparison.

(1) $$\begin{array}{r} PP\%=\left(\frac{\text{Corrected OD}450\text{ test sample}}{\text{Corrected OD}450\text{ positive control}}\;\times \;100\right)-10 \end{array}$$

### Statistical Analyses

Concordance correlation coefficients (CCCs) were computed in order to first compare the results of the two fresh aliquots, then, secondly, to compare results of the frozen aliquots with the initial analysis. CCCs values of 1 indicate perfect concordance, values approaching 1 indicate excellent concordance, and values inferior or close to 0 reflect very poor concordance. Bland-Altman diagrams were plotted to determine the limits of agreement, mean differences (biases) and 95% confidence intervals between the initial analysis and the second or third measurements (9). The repeatability of ELISA assays using provincial authorities' cut-off (PP% ≥ 15%) and manufacturer's recommended cut-off value (PP% ≥ 35%) was evaluated with Cohen's kappa (κ) as the measure of agreement. Before assessing kappa, we investigated whether the PP% values classified the same proportions of samples as positive using the McNemar's χ^2^ test in order for kappa assessment to be of value. Cohen's kappa (κ) were then calculated to assess the repeatability of the test to classify a sample as positive or negative. κ values were interpreted according to Landis and Koch's interpretation's guidelines (10). The data were stored in a Microsoft Access database and edited using version 9.4 of the SAS Software. CCCs analyses were performed using version 12 of the Stata Statistical Software (StataCorp LP, College Station, TX, USA), Concordance correlation and Bland-Altman plots were obtained using Excel, and Kappa analyses were performed for mixed population (mixture of positive and negative samples) using the EpiTools epidemiological software (https://epitools.ausvet.com.au/comparetwotests).

## Results

### Samples Analyzed

Each herd was sampled on average 2.9 times (range: 1 to 8 times). Out of the 845 bulk milk samples collected, 845 aliquots preserved at 4°C could be submitted to the initial ELISA analysis. They were analyzed on average within 6.7 days (range: 0 to 14 days) from sampling. Seventy-three percent (*n* = 616) of the 845 samples had their first analysis within the 5 days following the sampling. Twenty one percent (*n* = 176) were tested no more than 6 to 10 days after the sampling. The second ELISA analysis could be conducted on 845 aliquots. These second fresh samples were tested after an additional overnight-refrigeration at 4°C.

Regarding the frozen samples, 413 aliquots were frozen for about 2 weeks (mean 13.5 days; range: 13 to 14 days). Among these, 384 aliquots were included to the third ELISA analysis, while 29 could not be analyzed due to coagulation after thawing. The majority of the remaining 432 aliquots were frozen for about 4 weeks (mean 26.5 days; range: 25 to 28 days) and all of these could be analyzed.

### Concordance Between Fresh and Frozen Milk Samples

**Figure 1:**
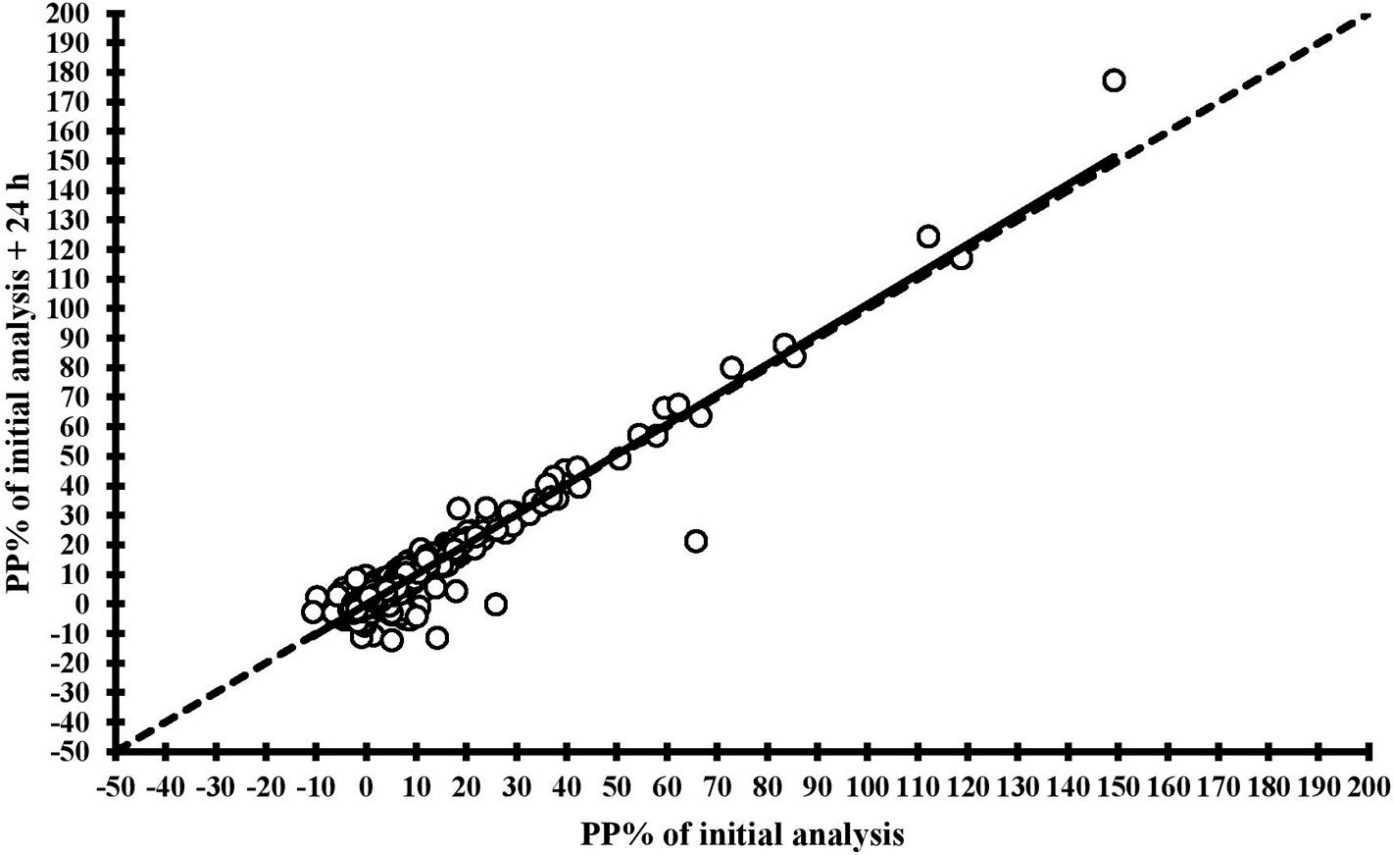
Concordance correlation plots comparing ELISA results for antibodies against *Salmonella* Dublin obtained from two different analyses of the same fresh bulk milk samples (analyses conducted 24 h apart after a 4°C overnight conservation), with regression lines (solid line) and lines of perfect concordance (dashed line).

The level of agreement between the two fresh samples and the fresh samples vs. frozen samples, are illustrated in Figure 1. Briefly, an agreement corresponding to a CCC of 0.96 (95% CI: 0.96, 0.97) was observed between the two fresh samples, and regression and perfect concordance lines were almost undistinguishable. Similarly, comparison of ELISA results of fresh samples vs. 13–14 days-frozen samples and 25–28 days-frozen samples indicated excellent concordance (Figures S2A,B). The corresponding CCCs values were 0.97 (95% CI: 0.97, 0.98) and 0.94 (0.93, 0.95), respectively.

### Biases and Limits of Agreement

**Figure 2:**
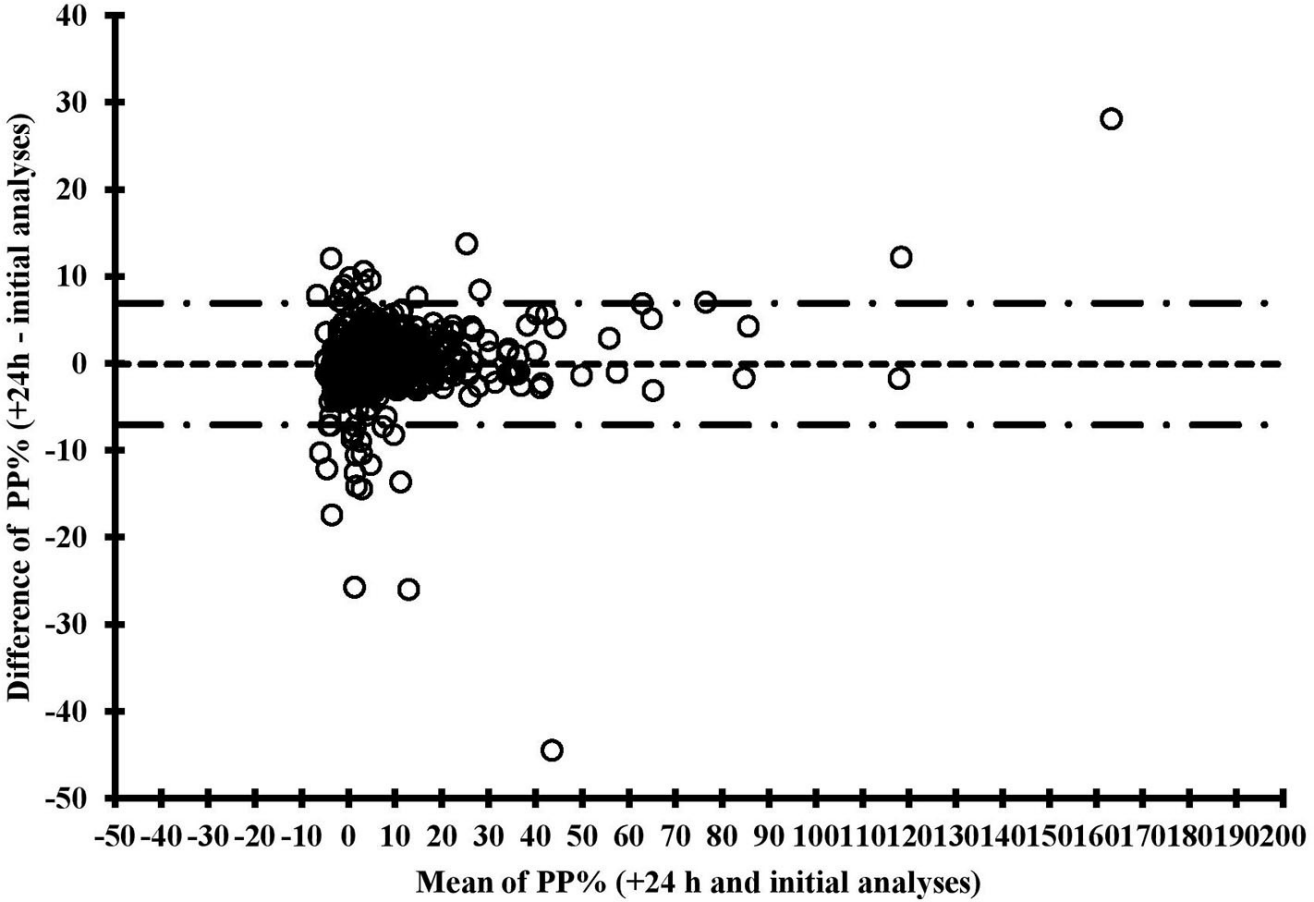
Bland-Altman plots for a *Salmonella* Dublin ELISA test comparing two different analyses of the same fresh bulk milk samples (analyses conducted 24 h apart after a 4°C overnight conservation), with mean difference between analyses (dashed line), and lower and upper limits of agreement (dashed and dotted lines).

The second fresh ELISA analysis underestimated by −0.1 percentage points (95% CI: −0.3, 0.1) the PP% values of the initial analysis (Figure 2). Conversely, the Bland-Altman plots obtained with frozen samples revealed overestimation by 0.1 (96%CI: −0.3, 0.5), which was not significant, and 0.4 (95% CI: 0.1, 0.8) percentage points on average, for 13–14 and 25–28 days of freezing, respectively (Figures S3A,B). For all comparisons, we observed larger PP% values variation (i.e., larger PP% differences) at lower mean PP% values, indicating greater variability of the assay in samples yielding low PP% values (Figure 2, Figures S3A,B). The number of observations with high PP% values was limited, however, thus precluding a thorough evaluation of PP% variation in samples yielding high PP% values. Finally, the limits of agreement obtained for 25–28 days-frozen samples was slightly larger than that of 13–14 days frozen samples (−7.1 to 8.0% vs. −6.9 to 7.1%).

### Repeatability of ELISA Assays for Determining Herd Status

The McNemar's χ^2^ test confirmed that, for all comparisons, when using same cut-off, the same proportions of samples were classified as positive in the two subsequent analyses (*P*-values ranging from 0.18 to 1.00), thus supporting use of the kappa statistic. Classification of the herds according to the two fresh samples analyses is presented in Table 1. Using the initial fresh samples analyses, the PP% ≥ 15% cut-off was leading to the classification of 98 herds (11.6%) as positive. The ≥35% cut-off was classifying 27 herds (3.3%) as positive. Almost perfect agreement (κ: 0.90) was observed between the two analyses, no matter the threshold used (Table 1). Very similar measures of agreement were observed when comparing the initial fresh samples analyses to the analyses conducted on samples frozen for 13–14 days. When freezing for 25–28 days, however, agreement with the initial fresh samples analyses was slightly lower for the PP% ≥ 15% cut-off (κ: 0.76; 95% CI: 0.67, 0.86).

**Table 1:** Contingency table of test results obtained from initial analysis, overnight refrigerating, freezing for 13 to 14 days, and freezing for 25 to 28 days analyses for assessment of the *Salmonella* Dublin ELISA assay repeatability using provincial authorities' cut-off (PP% ≥ 15%) and manufacturer's recommended cut-off (PP% ≥ 35%).

**Cut-off**	**Initial analysis**	**Positive**	**Negative**	**McNemar's χ^2^*P*-value**	**Kappa (95% CI)**
		Analysis +24 h			
PP% ≥ 15%	Positive	89	9	1.00	0.90 (0.86, 0.95)
	Negative	8	739		
PP% ≥ 35%	Positive	23	4	0.37	0.90 (0.81, 0.99)
	Negative	1	817		
		Analysis +13–14 days			
PP% ≥ 15%	Positive	40	7	0.18	0.89 (0.81, 0.96)
	Negative	2	335		
PP% ≥ 35%	Positive	10	1	0.48	0.91 (0.78, 1.00)
	Negative	1	372		
		Analysis +25–28 days			
PP% ≥ 15%	Positive	42	8	0.29	0.76 (0.67, 0.86)
	Negative	14	368		
PP% ≥ 35%	Positive	15	1	1.00	0.91 (0.80, 1.00)
	Negative	2	414		

**Figure 3:**
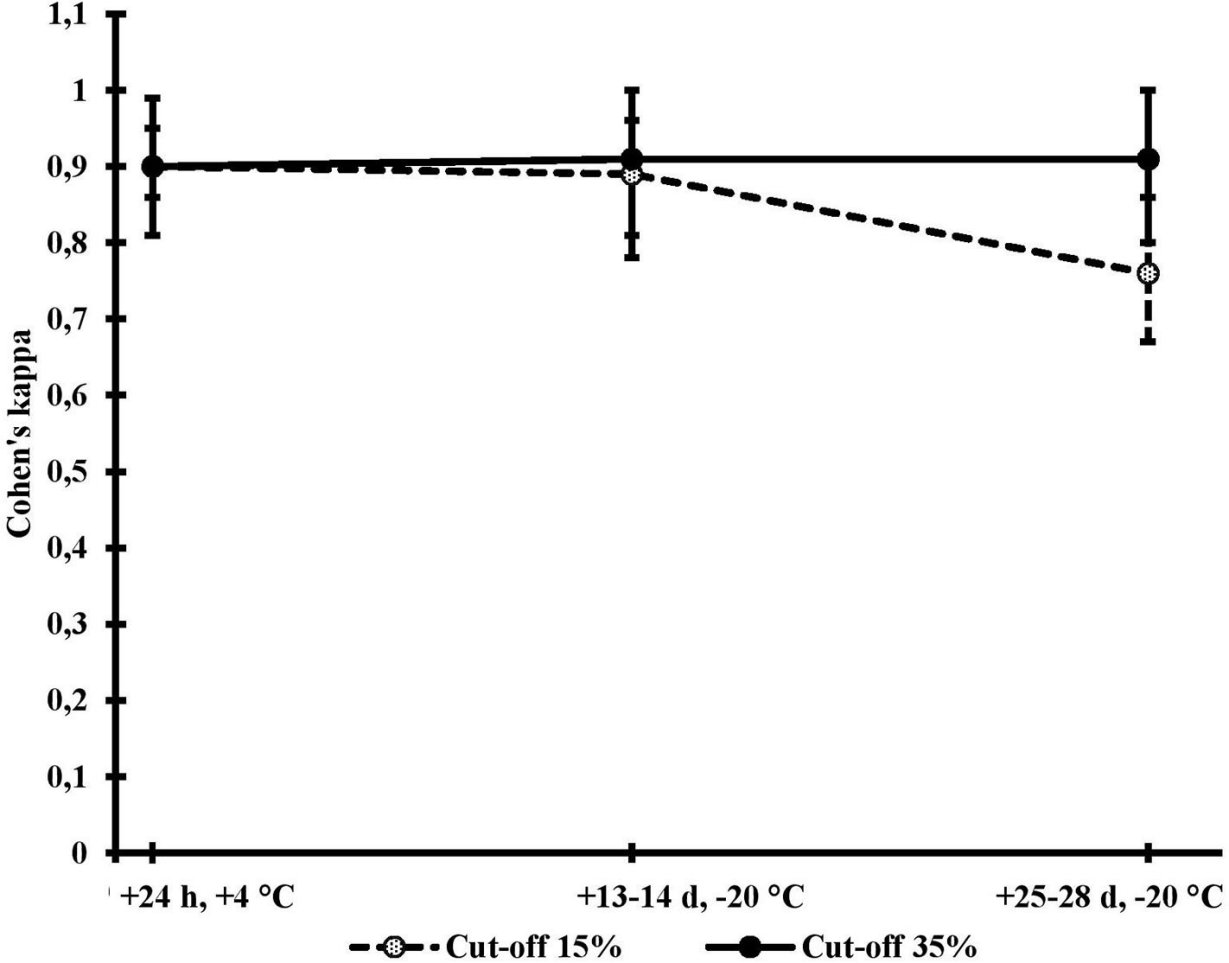
Measures of agreement (Cohen's kappa) with confidence intervals between two ELISA results for antibodies against *Salmonella* Dublin obtained from the same fresh bulk milk samples analyzed twice at 24 h intervals 13–14 days and 25–28 days and using for interpretation the manufacturer's cut-off (35%; solid line) or our provincial authorities' cut-off (15%; dashed line).

Figure 3 summarizes agreement between analyses for the two cut-offs. The agreement was almost perfect when the ELISA results were interpreted using the cut-off value PP% ≥ 35%, regardless of freezing duration. When the ELISA results were analyzed using the cut-off values PP% ≥ 15%, the agreement decreased with increasing freezing time.

## Discussion

The aim of this study was to determine the repeatability of PrioCHECK™ *Salmonella* Ab Bovine Dublin when used in the context of a surveillance program that would include various samples handling conditions. The ELISA PP% values of two fresh aliquots from 845 bulk milk samples were compared at 24 h of interval. The assay variability of plausible freezing for 2 and 4 weeks on ELISA results was also investigated.

The CCC analyses indicated values close to 1 indicating excellent concordance between fresh and frozen bulk milk samples. Few studies have investigated the repeatability of ELISA milk-based tests under stressors. Evidence of biologically negligible effect of individual milk handling stressors (heating, freezing, thawing and re-freezing for 4 weeks) on a parasite ELISA test results has been described in a Canadian study (11). The CCCs values obtained in our study (0.94–0.97) were coherent with the range of CCC values of 0.65–0.99 reported in the latter study for all possible stressors combinations with interquartile ranged from 0.91 to 0.98 for all CCCs.

Bland-Altman plot results revealed a non-significant decrease of 0.1% following the overnight storage. We observed increases of 0.1% (non-significant) and 0.4% percentage-points after freezing for 13–14 days and 25–28 days, respectively. Similar slight increase following storage at −20°C for 14 days and 28 days was observed on pregnancy-associated glycoproteins ELISA results for milk using multivariable analysis (12). Although, direct comparison between the two studies cannot be made, some of the storage temperatures and durations used in this study are comparable to the Wynands et al. study. The reasons for such changes remain unknown. However, hypotheses that the protein concentration could have been increased in milk sample due to evaporation during freezing or that the denaturated proteins could cross-react with the ELISA enzymes have been suggested by the authors.

In the current study, we did not investigate whether the number of days between sample collection and first analysis could affect the repeatability of the test. In our study, 69 and 79% of samples had a first analysis conducted within, respectively, 3 and 7 days of collection. There was, therefore, relatively little variation in our dataset in number of days between collection and first analysis, precluding the investigation of the effect of this parameter on the test repeatability. The repeatability could be different in settings where longer withholding times are used prior to the first analysis.

The slight increase in ELISA PP% values for the 25–28 days frozen samples compared with the initial values was statistically significant. The Bland-Altman analysis is a good approach for authorities to assess by how far the bias between frozen and initial samples is acceptable and what limits of agreement are tolerable (13). The PP% value of the milk sample could be corrected by removing 0.4% to the frozen sample PP% measure for samples frozen for 25–28 days. With the current study design, however, we cannot predict if this bias would increase with longer freezing duration.

Concerning the classification of a herd as *S*. Dublin positive or negative, kappa analyses showed a greater disagreement between initial and frozen samples when ELISA results were interpreted using the authorities' cut-off PP% ≥ 15% compared to manufacturer's recommended cut-off PP% ≥ 35%. This could be due to the observed higher variation of the assay at lower PP% values.

In conclusion, our study showed that ELISA analyses using PrioCHECK™ *Salmonella* Ab Bovine Dublin in fresh bulk milk samples and 2 to 4 weeks-frozen samples were almost identical. The measured changes in PP % values were minimal. The test had good repeatability and frozen bulk milk samples could yield repeatable results. A greater disagreement for classifying herds as positive or negative was observed as length of freezing increased when interpreting ELISA results with a PP% ≥ 15% cut-off. Our future research will now focus on determining the diagnostic accuracy of this milk ELISA test for determining the herd-level *S*. Dublin status when used on bulk milk samples and for different cut-offs and sampling strategies.

## Data Availability Statement

The raw data supporting the conclusions of this article will be made available by the authors, without undue reservation.

## Ethics Statement

The animal study was reviewed and approved by Université de Montréal's Research Ethic Committee (18-Rech-1943). Written informed consent was obtained from the owners for the participation of their animals in this study.

## Author Contributions

SD conceived the project. MU and SD designed the study, analyzed the data, and prepared the manuscript. M-HC and IM organized the sampling. KM and JG conducted laboratory analyses. All authors reviewed the manuscript.

## Conflict of Interest

MU and SD are members of Op+lait which is a multi-universities independent research group funded by competitive grants obtained from the provincial government. M-HC, KM, JG, and IM were employed by Lactanet Laboratories. The remaining authors declare that the research was conducted in the absence of any commercial or financial relationships that could be construed as a potential conflict of interest.

## Supplementary Material

Figure S1 Flow chart describing flow of samples prior to analyses.

Figure S2 Concordance correlation plots comparing fresh samples with 13–14 days-frozen samples *Salmonella* Dublin ELISA results **(A)**, and fresh samples with 25–28 days-frozen samples *Salmonella* Dublin ELISA results **(B)**, with regression lines (solid line) and lines of perfect concordance (dashed line).

Figure S3 Bland-Altman plots of the fresh samples vs. 13–14 days-frozen samples ELISA results **(A)**, and fresh samples vs. 25–28 days-frozen samples ELISA results **(B)**, with mean difference between analyses (dashed line), and lower and upper limits of agreement (dashed and dotted lines).

## References

[1] MAPAQ Ministry of Agriculture, Fisheries and Food of Quebec (Ministère de l'Agriculture, des Pêcheries et de l'Alimentation du Québec) (2018). Available online at: https://www.mapaq.gouv.qc.ca/fr/Productions/santeanimale/maladies/soussurveillance/Pages/Salmonella-Dublin.aspx (accessed April 23, 2020)

[2] Davison HC Smith RP Pascoe SJ Sayers AR Davies RH Weaver JP. Prevalence, incidence and geographical distribution of serovars of *Salmonella* on dairy farms in England and Wales. Vet Rec. (2005) 157:703–11. 10.1136/vr.157.22.703 16311384

[3] Nielsen LR. Review of pathogenesis and diagnostic methods of immediate relevance for epidemiology and control of *Salmonella* dublin in cattle. Vet Microbiol. (2013) 162:1–9. 10.1016/j.vetmic.2012.08.003 22925272

[4] O'Reilly KJ Smith CE McMahon DA Wilson AL Robertson JM. Salmonellae in cattle and their feedingstuffs, and the relation to human infection. A report of the joint working party of the veterinary laboratory services of the Ministry of Agriculture, Fisheries and Food, and the Public Health Laboratory Service. J Hyg. (1965) 63:223–41. 10.1017/S0022172400045125 14308353 PMC2134652

[5] Watson DL. Immunological functions of the mammary gland and its secretion–comparative review. Aust J Biol Sci. (1980) 33:403–22. 10.1071/BI9800403 7004419

[6] Smith BP Oliver DG Singh P Dilling G Martin PA Ram BP Detection of *Salmonella* dublin mammary gland infection in carrier cows, using an enzyme-linked immunosorbent assay for antibody in milk or serum. Am J Vet Res. (1989) 50:1352–60. 2675696

[7] Hoorfar J Lind P Bitsch V Evaluation of an O antigen enzyme-linked immunosorbent assay for screening of milk samples for *Salmonella* dublin infection in dairy herds. Can J Vet Res. (1995) 59:142–8. 7648527 PMC1263752

[8] Nyman AK Agren EC Bergstrom K Wahlstrom H. Evaluation of the specificity of three enzyme-linked immunosorbent assays for detection of antibodies against *Salmonella* in bovine bulk milk. Acta Vet Scand. (2013) 55:5. 10.1186/1751-0147-55-5 23360615 PMC3639889

[9] Bland JM Altman DG. Statistical methods for assessing agreement between two methods of clinical measurement. Lancet. (1986) 1:307–10. 10.1016/S0140-6736(86)90837-8 2868172

[10] Landis JR Koch GG. The measurement of observer agreement for categorical data. Biometrics. (1977) 33:159–74. 10.2307/2529310 843571

[11] Vanderstichel R Dohoo I Stryhn H. The impact of milk handling procedures on *Ostertagia ostertagi* antibody ELISA test results. Vet Parasitol. (2010) 169:204–8. 10.1016/j.vetpar.2009.12.006 20053502

[12] Wynands EM LeBlanc SJ Kelton DF. Short communication: the effect of storage conditions and storage duration on milk ELISA results for pregnancy diagnosis. J Dairy Sci. (2017) 100:9781–6. 10.3168/jds.2017-12799 28987575

[13] Bland JM Altman DG Statistical methods for assessing agreement between two methods of clinical measurement. Int J Nurs Stud. (2010) 47:931–6. 10.1016/j.ijnurstu.2009.10.001 2868172

