# Supplementary figures and images for: Repeatability of a Commercially Available ELISA Test for Determining the Herd-Level Salmonella enterica subsp. enterica Serovar Dublin Status in Dairy Herds Using Bulk Milk
Source: Front Vet Sci. 2020 Jul 10;7:401. doi: 10.3389/fvets.2020.00401 (PMC7366779; doi:10.3389/fvets.2020.00401)

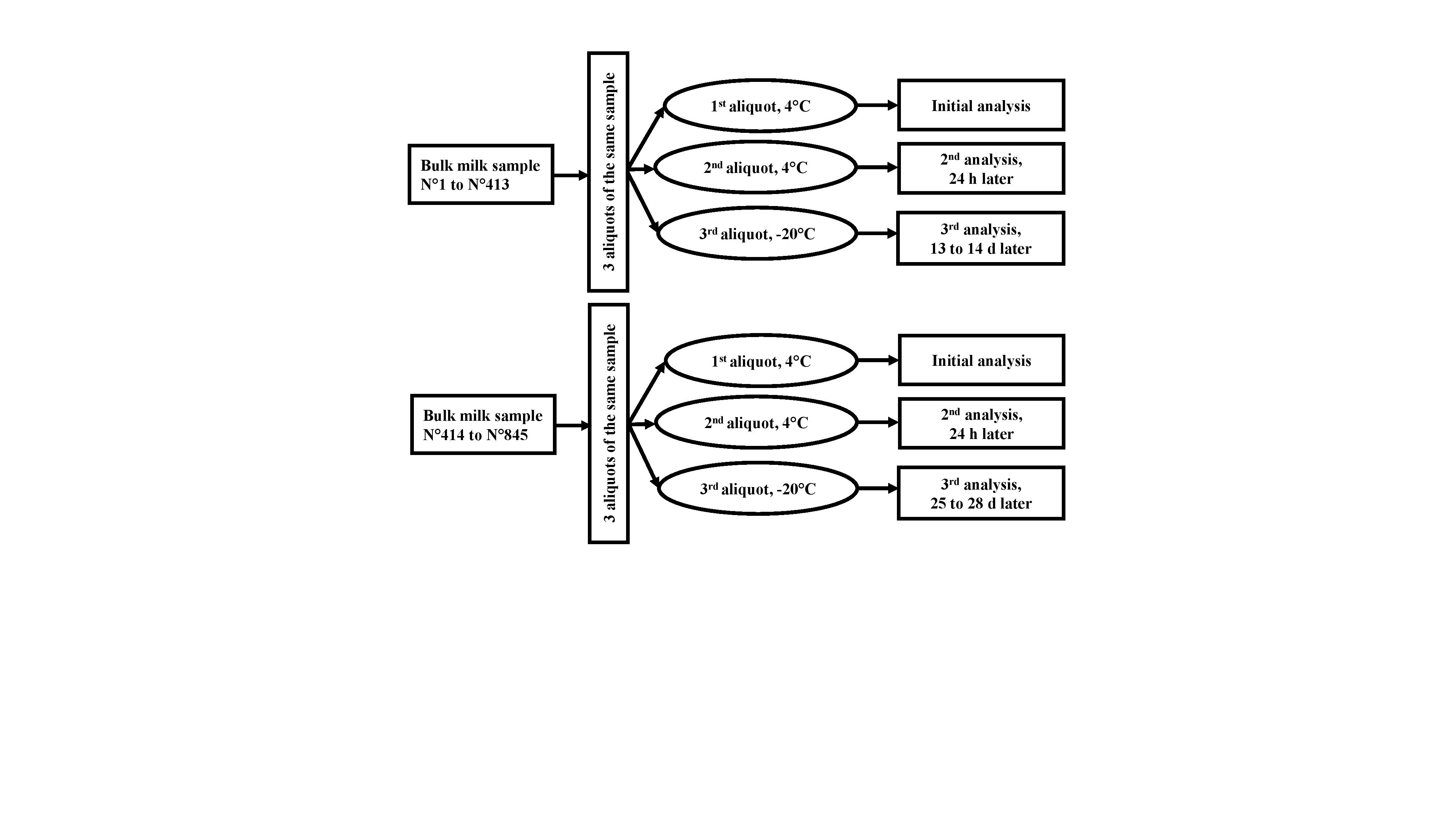

Supplement: Figure S1 — Flow chart describing flow of samples prior to analyses. [file Image_1.jpeg]

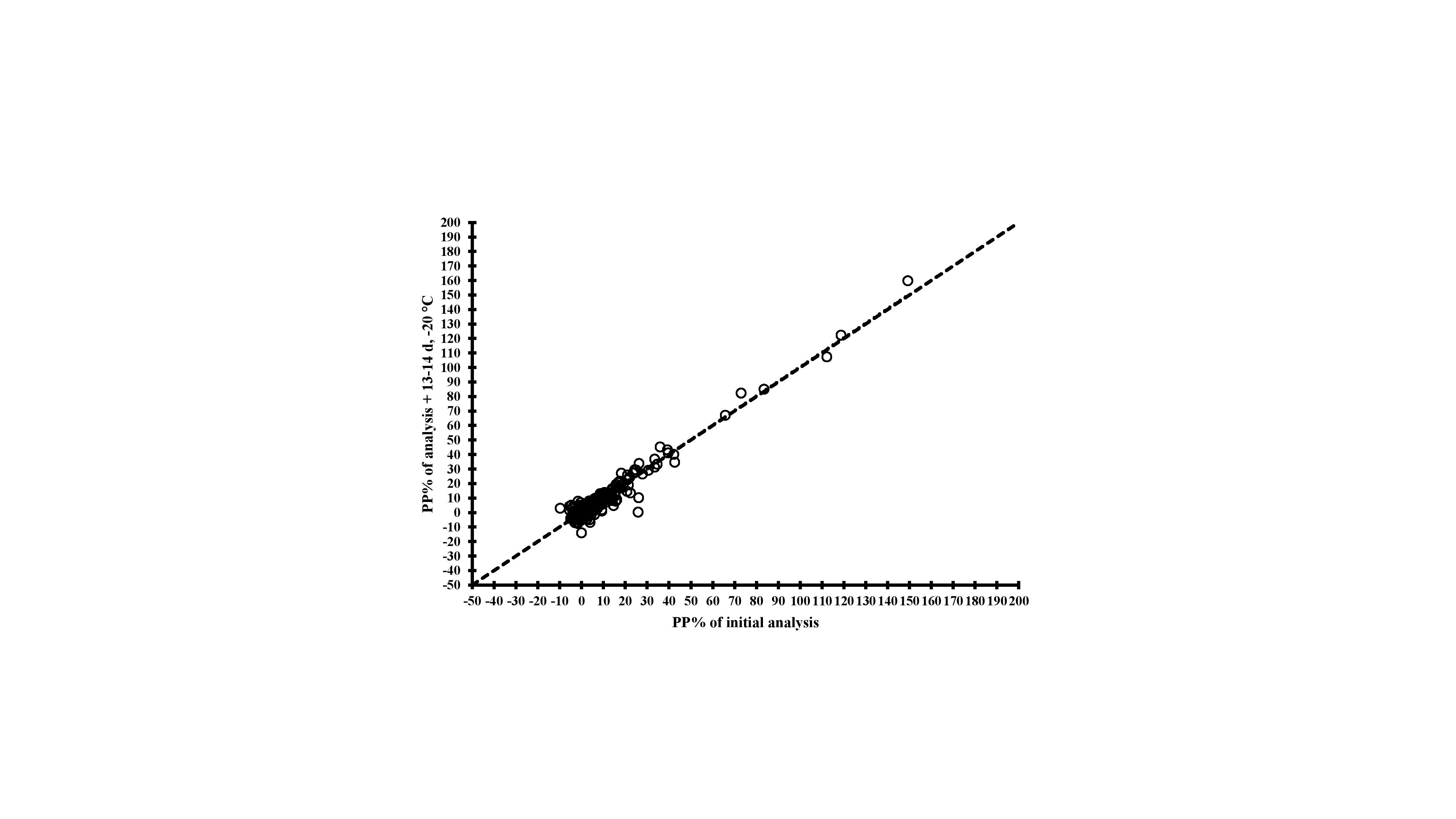

Supplement: Supplementary file 2 [file Image_2.jpeg]

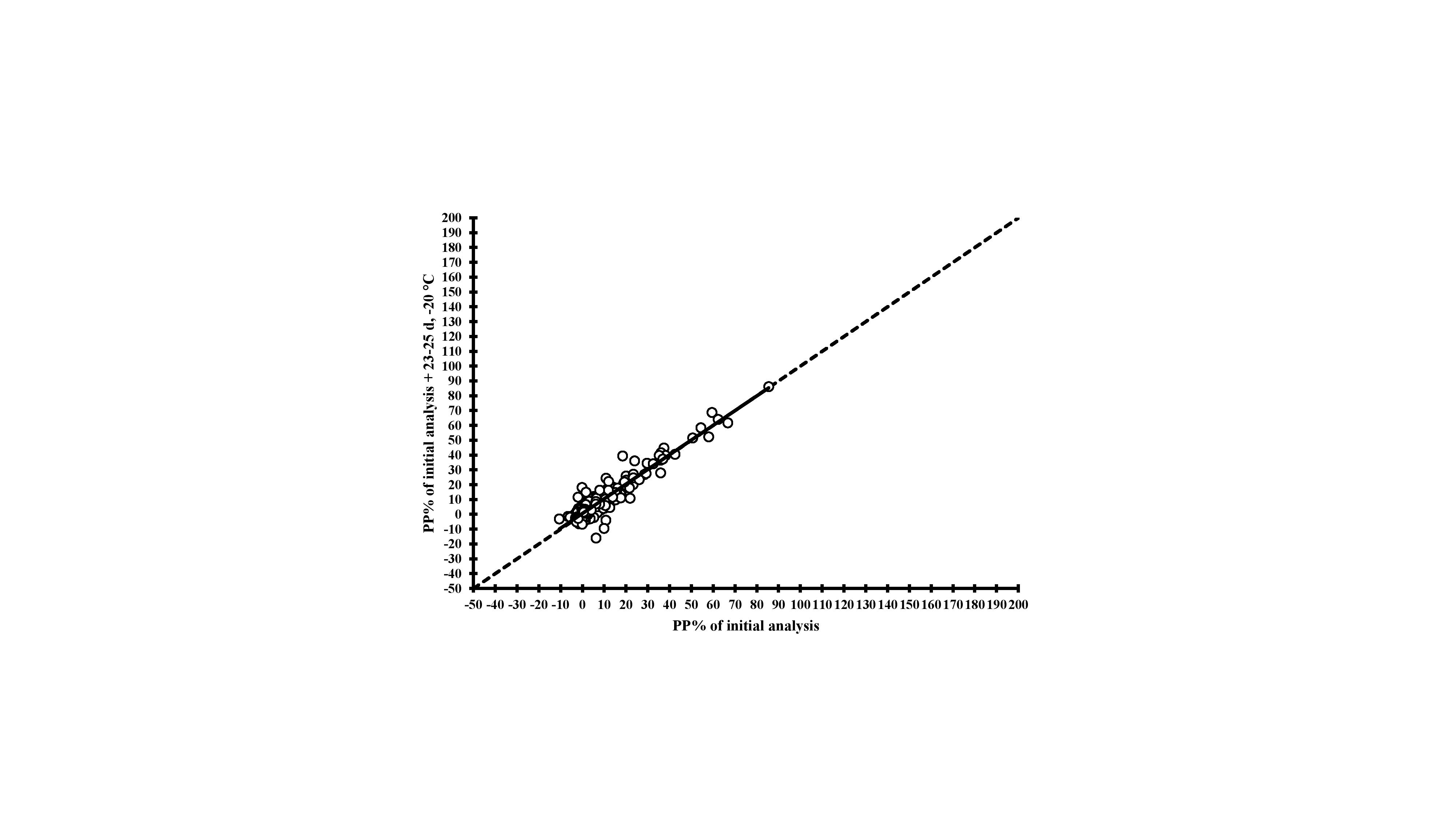

Supplement: Figure S2 — Concordance correlation plots comparing fresh samples with 13–14 days-frozen samples Salmonella Dublin ELISA results (A), and fresh samples with 25–28 days-frozen samples Salmonella Dublin ELISA results (B), with regression lines (solid line) and lines of perfect concordance (dashed line). [file Image_3.jpeg]

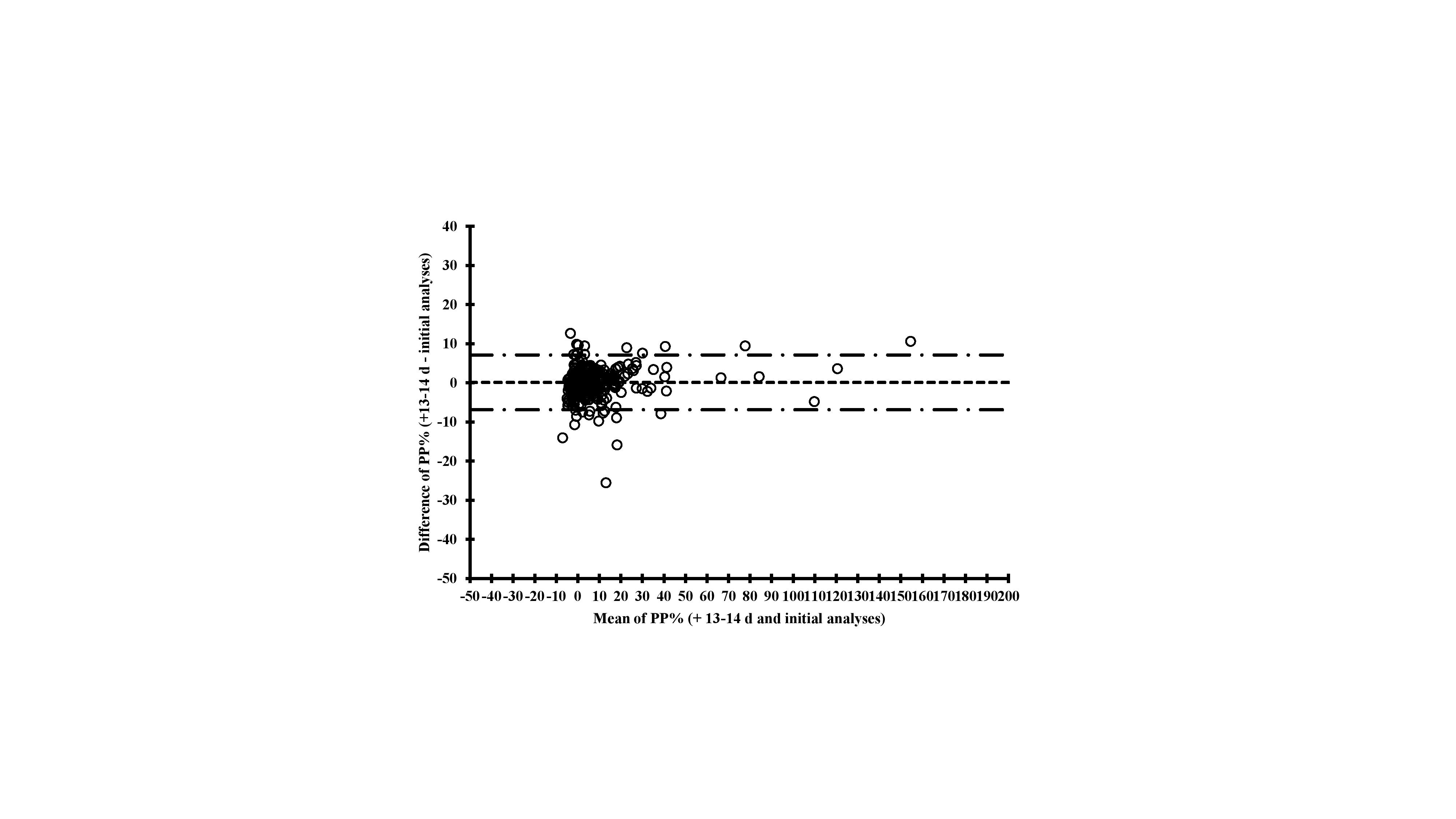

Supplement: Supplementary file 4 [file Image_4.jpeg]

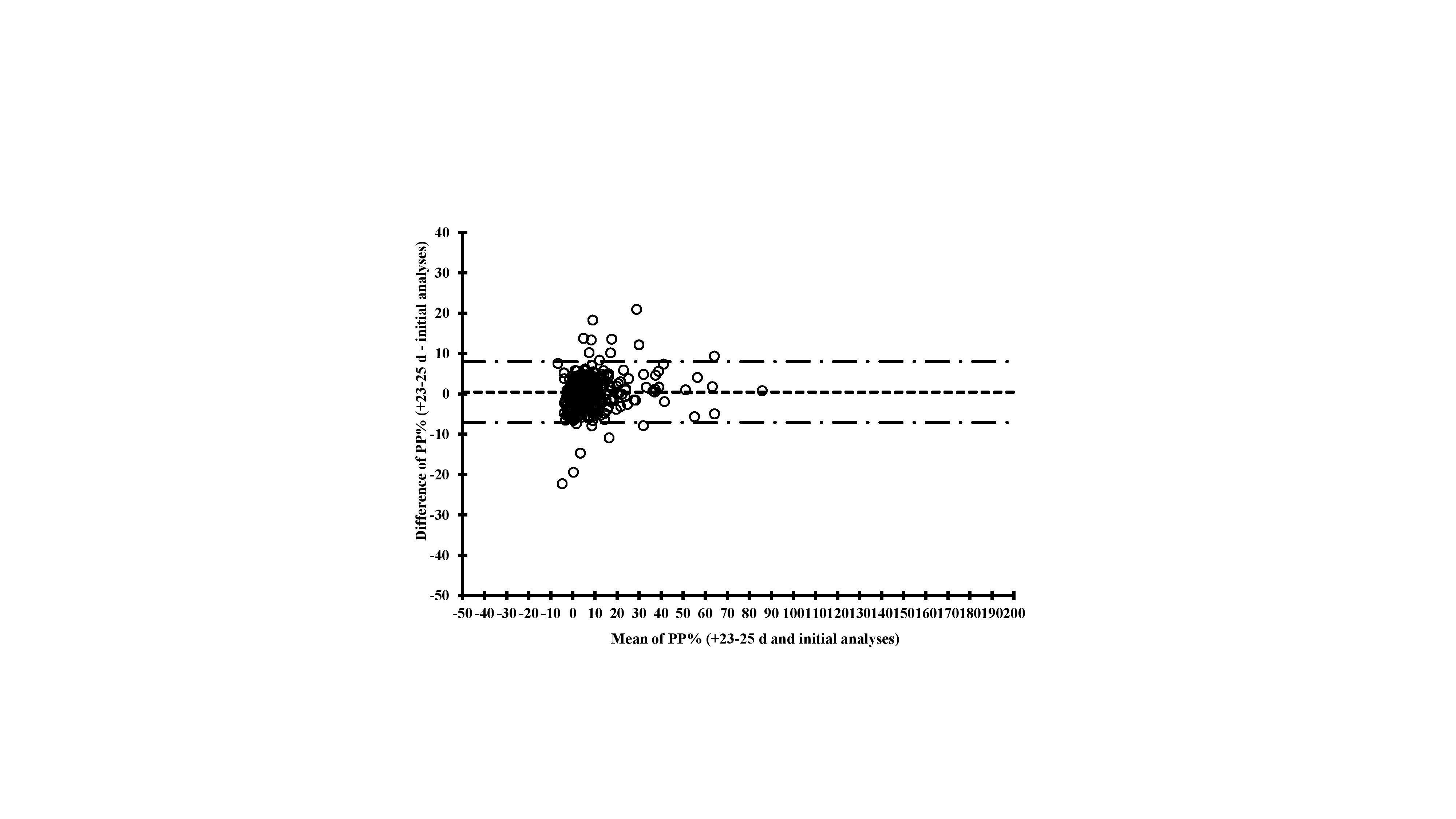

Supplement: Figure S3 — Bland-Altman plots of the fresh samples vs. 13–14 days-frozen samples ELISA results (A), and fresh samples vs. 25–28 days-frozen samples ELISA results (B), with mean difference between analyses (dashed line), and lower and upper limits of agreement (dashed and dotted lines). [file Image_5.jpeg]
